# The Heterogeneous Complexity of Myeloid Neoplasm: Multi-Level Approaches to Study the Disease

**DOI:** 10.3390/cancers15051449

**Published:** 2023-02-24

**Authors:** Hussein Awada, Valeria Visconte

**Affiliations:** Department of Translational Hematology and Oncology Research, Taussig Cancer Institute, Cleveland Clinic, Cleveland, OH 44106, USA

Myeloid neoplasms (MNs) include a spectrum of bone marrow malignancies that result from the clonal expansion and arrest of differentiation of myeloid progenitor cells [1]. MNs account for around 25% of all hematological malignancies and typically arise in older patients who are in their seventh or eight decade as they accumulate genetic alterations throughout their lifetime [2]. Perturbation of normal genetic and epigenetic regulation is mostly due to the disruption of key cellular processes such as self-renewal, proliferation, and differentiation [1]. The rise of population aging and exposure to environmental carcinogens have been attributed to triggering bone marrow insults [3,4]. Myelodysplastic syndromes (MDS), acute myeloid leukemia (AML) and myeloproliferative neoplasm (MPN) remain the most frequently encountered MNs and hence are the subject of the foremost research efforts as well. However, despite the mortality risks of these diseases, their courses are highly variable in terms of response to therapies and survival, which range from weeks to several years.

While novel clinical markers were long considered in the past to better characterize these malignancies, the heterogeneous genetic nature of these disorders has left us with more questions. Therefore, seeking explanations behind the underlying pathogenetic processes driving clonal trajectories has prompted us to shift our focus more toward the understanding of the landscape of genetic aberrations instigating these malignancies [5]. Technologies to detect genetic defects have been largely used over the years, spanning from the gold standard karyotype analysis and fluorescence in situ hybridization to plasmid cloning and sequencing, single nucleotide polymorphism (SNP) arrays, comparative genomic hybridization arrays (CGH) and classical bidirectional sequencing [6,7]. The latter has been replaced by large-volume DNA segment amplified sequencing via next-generation sequencing (NGS), either through whole exome and genome or targeted deep sequencing [8,9].

The introduction of RNA sequencing (RNA-seq) via NGS further added new dimensions by which sequencing techniques detect polymorphisms spanning from differential expression or alternative splicing (AS). By converting extracted messenger RNAs (mRNA) into complementary DNA (cDNA), RNA-seq provides more accurate differential quantification of highly or lowly expressed genes while examining the function of cellular transcriptome through changes in gene expression, AS, or isoforms [10]. However, RNA-seq may obscure small yet significant differences between individual cell subsets when large amounts of cells are sequenced. Such differences may be critical in the early processes of clonal evolution of myeloid disorders into more aggressive diseases, disease relapse, as well as the assessment of minimal residual disease [11]. Further integration of single-cell DNA sequencing (scDNA-seq) to study the methylome complements scRNA-seq by uncovering methylation patterns influencing the levels of expression detected by scRNA-seq in leukemia cells at single-cell level. Further insights on gene regulatory landscapes may also be inferred by the assay for transposase-accessible chromatin using ATAC-seq which isolates and quantifies non-coding DNA regions. Hence, it provides a wider view of possible targetable active genomic areas of transcriptional influence and subsequent myeloid disease evolution [12].

Diagnostic NGS mutational panels are today an integral part of the management of myeloid disorders as they have been included in diagnostic tests. Along with the techniques mentioned above, diagnostic NGS has yielded the identification of several classes of leukemogenic drivers. Among others, the most relevant include epigenetic DNA methylation regulators (e.g., ASXL1, DNMT3A, EZH2, IDH1, IDH2, TET2), tumor suppressors (e.g., TP53), signaling pathways activators (e.g., FLT3, KRAS, NRAS, JAK2), transcription factors (e.g., CEBPA, ETV6, RUNX1), splicing factors (e.g., SF3B1, SRSF2, U2AF1) as well as shuttling proteins (e.g., NPM1) [1,13,14,15]. The hallmark of unmasking these leukemogenic mutations is to determine the unique genetic profile dictating the course and prognosis of each patient. Indeed, molecular mutations set the groundwork for improved classifications as reflected in the 2022 European Leukemia Net (ELN) recommendations which have further incorporated mutations in *BCOR*, *EZH2*, *SF3B1*, *SRSF2*, *STAG2*, *U2AF1* and *ZRSR2* genes into its adverse risk category of AML on top of their 2017′s recommendations for inclusion of *ASXL1*, *RUNX1* and *TP53* [16,17]. Moreover, the International Prognostic Scoring System (IPSS) and its revised version (IPSS-R) are now falling in favor of the molecularly upgraded IPSS-M which incorporates the lesions in 31 genes of confirmed independent impact on MDS prognosis [18]. The new mutation system divides MDS patients into six survival strata [18]. Similarly, a molecular version of the chronic myelomonocytic leukemia (CMML)-specific prognostic scoring system (CPSS-Mol) entailing *ASXL1*, *NRAS*, *RUNX1* and *SETBP1* mutations provides better clinical insights than the original CPSS [19]. Unveiling these mutations further serves as the cornerstone for new differential hematological entities, including benign conditions such as clonal hematopoiesis of indeterminate potential (CHIP) and clonal cytopenia of undermined significance (CCUS).

However, perhaps the biggest clinical benefit of understanding the genetics and epigenetics of MNs pertains to identifying targetable mutations whose inhibition may improve disease outcomes. As more and more mutations are discovered, targeted panels are further expanding and thus leave us with more options for precision target therapy. Indeed, the identification of IDH1 and IDH2 as targetable mutations led to the introduction of the IDH1 inhibitors, Enasidenib and Ivosidenib, and the IDH2 inhibitor, Olutasidenib, which are now approved in full effect for refractory/relapsed MN [20].

As the use of modern genomic sequencing techniques has become the tested workhorse in characterizing new myeloid disease diagnoses, further advancement is still seeking to understand molecular interactions while also minimizing the role of human bias. In this context, the application of machine learning (ML) algorithms is becoming one approach to attempt simplification of complexity. As opposed to supervised learning, unsupervised learning prevents human bias and hence may contribute to increasing the accuracy of analyses by eliminating unnoticed errors perpetrated in prioritizing unrepresentative datasets, effect measurements and reporting [21]. Traditional unsupervised Artificial Intelligence (AI) techniques, especially Deep Learning (DL) algorithms, allow the integration of molecular signatures for disease subclassification, prognostication, and prediction of treatment response. More importantly, it allows the integration of multi-omics in one model (Figure 1).

This is the case of novel studies performed in our laboratory by combining genomic and transcriptomic data of MNs [22]. For example, DL techniques succeeded in the novel subcategorization of newly diagnosed AML patients regardless of their primary or secondary subtypes into four clusters of invariant molecular features and unique prognoses [23]. In this multicenter study of 6,788 AML patients, the unsupervised analysis resulted in 97% cross-validation accuracy, far superior to the 74% accuracy yielded when applying standard supervised analysis attempting to use molecular patterns to predict traditional path-morphologic classifications [23]. Similar unsupervised learning approaches have identified 14 distinct molecular clusters of clinical heterogeneity in MDS [24]. Each of these clusters has its unique pathobiological associations, treatment responses, and prognosis [24].

ML approaches are also capable of unmasking the morphological consequences of specific molecular signatures while also tracing the evolutionary origins of MNs [25]. In another study, 1079 MDS patient specimens were sequenced in order to define an association between molecular profiles and bone marrow morphologic characteristics and clinical traits [25]. Five unique morphological profiles with distinct clinical features were identified, among which profile 1 was mostly high-risk MDS while the low-risk disease predominant profiles 2, 3, 4 and 5 were characterized by pancytopenia, monocytosis, megakaryocytosis and erythroid dysplasia, respectively [25]. In turn, the low-risk MDS group was classified into eight genetic groups, which served as the basis for subsequent geno-morphologic combinations [25]. Six geno-morphologic signature associations were yielded and hence improved our understanding of the impact of genetic alterations on clinic-morphologic traits [25].

So far, the diagnosis of MN fairly relies on bone marrow studies, yet these studies are subject to inter-observer variability bias as the diagnosis might be challenging in certain scenarios, especially in patients with pancytopenia or minimal dysplasia [26]. Radakovich et al. instead developed an ML model for MN diagnosis based on genomic and clinical data only; thus, it can be used to empower diagnostic decision-making in cases of uncertainty. In this international multicenter study, a gradient-boosted DL strategy was adopted as the geno-clinical features of 2697 patients with MDS or one of the MPN disorders were used to train their model [26]. The final model retained 15 geno-clinical variables, including *JAK2*, *KRAS*, and *SF3B1* status, and then proved to accurately differentiate MDS, MPN subclasses, as well as benign conditions like CHIP, CCUS and Idiopathic cytopenia of undetermined significance with AUROC of 0.951 and 0.926 for the test and training cohorts, respectively [26].

Moreover, ML techniques may be helpful in developing personalized models that predict treatment response and thus aid in personalizing treatment selection according to each patient’s characteristics. Along with this line of research, Nazha et al. screened the genomic architecture of a cohort of 433 MDS patients with varying responses to the hypomethylating agents (HMA) azacitidine and decitabine [27]. By utilizing an unbiased ML recommender system, mutational signatures composed of two to three mutations in *ASXL1*, *BCOR*, *EZH2*, *NF1*, *RUNX1*, *SRSF2* and *TET2*, were identified as an association with HMA resistance [27]. The result had an accuracy rate of 87% and 93% in the training and validation cohorts, respectively [27]. Such finding is further enforced by the estimation of around 30% of HMA-treated MDS patients had at least one of the signatures, and thus using ML techniques could have accurately predicted HMA resistance and the need to consider other therapeutic options [27].

Despite its proven utility, the implementation of AI methods in the daily clinical setting certainly remains challenging. It requires physicians to understand AI basics, as well as institutions to be capable of implementing its networks in their medical records. The latter brings about several hurdles, including the need for electronic types of medical records in order for AI to easily recall the large volumes of patient data that are necessary for its proper functioning. In addition, having the appropriate logistics, such as experienced data scientists, dedicated clinician-scientists, availability of the required technological tools, the volume and adequacy of data used to develop AI models, as well as clinically meaningful needs and standards for AI development are all prerequisites for its successful implementation in clinics.

## Figures and Tables

**Figure 1:**
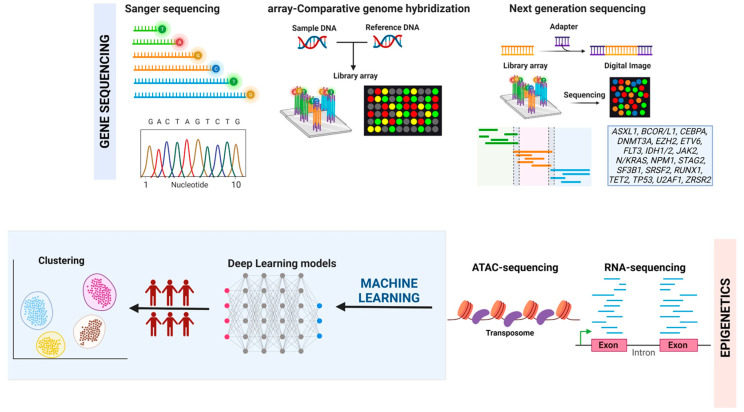
Integration of genomic and transcriptomic features using machine learning. Schematic representation of the possible combination of results derived from techniques used to study gene variations and RNA profiles including chromatin analysis. The figure was created using BioRender.com.
